# COVID-19 Effect on Education in Pediatric Dentistry

**DOI:** 10.3389/fped.2021.666501

**Published:** 2021-09-06

**Authors:** Sreekanth Kumar Mallineni, Sivakumar Nuvvula, Virinder Goyal, Figen Seymen

**Affiliations:** ^1^Department of Preventive Dental Science, College of Dentistry, Majmaah University, Al-Majmaah, Saudi Arabia; ^2^Department of Pediatric and Preventive Dentistry, Saveetha Dental College and Hospital, Saveetha Institute of Medical and Technical Sciences, Saveetha University, Chennai, India; ^3^Department of Pediatric and Preventive Dentistry, Narayana Dental College and Hospital, Nellore, India; ^4^Department of Pediatric and Preventive Dentistry, Gurunanak Dev Dental College, Patiala, India; ^5^Department of Pediatric Dentistry, Faculty of Dentistry, Istanbul University, Istanbul, Turkey

**Keywords:** dental education, pediatric dentistry, education methods, COVID-19, children

## Highlights

- Evolution of education in pediatric dentistry from classroom teaching to virtual teaching.
- Online education in pediatric dentistry through webinars.
- Introduction of the concept of teledentistry in pediatric dentistry for the benefit of children and students attending pediatric dentistry postings.
- Evolution of new strategies for students, academicians and clinicians attending pediatric dentistry clinics by various international associations around the globe.

## Introduction

Experience with child patients is mandatory for dental students to improve their clinical abilities, identify, and manage children's dental problems. There was a suspension of on-campus learning due to institutions' closure, eliminating real-time clinical sessions during the coronavirus disease (COVID-19) pandemic. Hence, it is crucial to develop alternative methods for education in light of clinical closures. A French group of authors (1) suggested temporary modification in clinical care (academic and private practice) and curriculum through incorporating video conferences, e-learning sessions, and webinars. To deal with educational issues with the child patient experience, the introduction of pediatric dentistry or the first dental visit provides a prospective elucidation to dental education, especially in the present COVID-19 pandemic outbreak. The recent COVID-19 pandemic impact on dental education had have been discussed by various authors from different parts of the world in the contemporary literature (2–5). Shah (6) from Pakistan opined that it is essential to implement specific protocols to minimize the transmission of COVID-19 infection to patients from other patients or medical tools and equipment. Kochhar et al. (7) from north India recommended that a customized approach is necessary to safeguard children and their accompanying family members and health care professionals in the dental operatory from the novel Coronavirus. Bahramian et al. (8) from Iran reported that social media's effect on updating knowledge on protocols and other necessary information on COVID-19 was beneficial for a worldwide approach to safety in the educational setting. A recent editorial by Paglia (9) from Italy opined that pediatric dentists should rethink and review the daily clinical practice concerning airborne viral safety. However, there is a paucity of information on the impact of COVID-19 on education in pediatric dentistry. Furthermore, there is no discussion about COVID-19's impact on students attending pediatric dentistry clinical experiences. Thus, there is a need to discuss the adaptations required to enhance dental student (undergraduate and postgraduate) pediatric dentistry learning.

## Discussion

**Figure 1:**
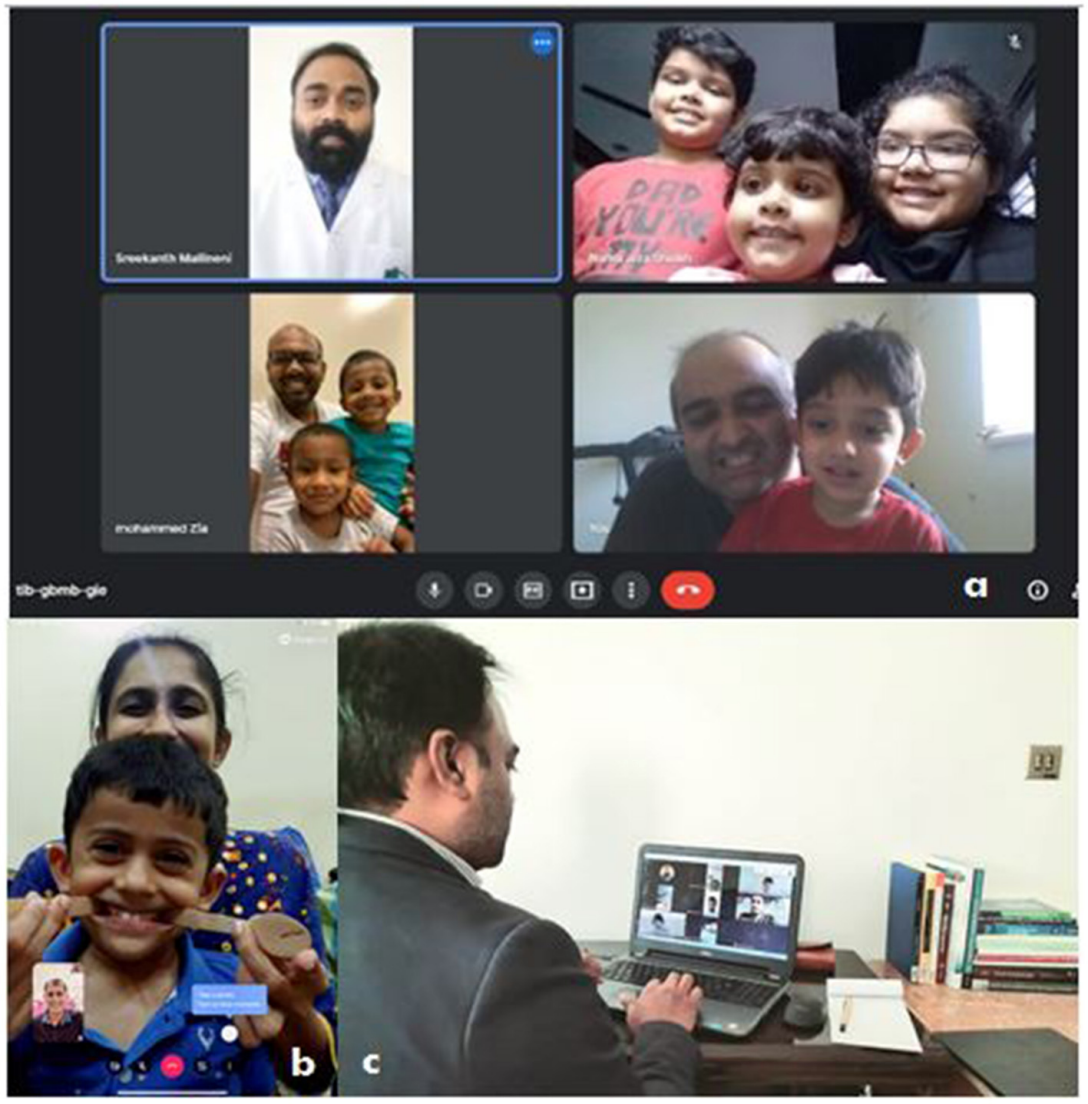
**(a)** Virtual patient consultations, **(b)** parental guidance, and **(c)** student meetings during COVID-19 pandemic outbreak on pediatric dentistry.

Pediatric dentists can implement virtual appointments for pediatric dental consults, and dental students can participate in these meetings with faculty members. Numerous sources validate video conferencing with the child and parent during virtual patient care meetings (Figures 1a,b). This virtual communication allows dental students to learn fundamental pediatric dental examination and preventive strategy concepts improving their knowledge in the absence of in-person clinical appointments (Figure 1c). Telecommunication with children and parents is recommended for pediatric dentists during pandemic outbreaks due to the increased risk of viral transmission (10). It is imperative to implement strict guidelines and protocols in the offices and institutions dealing with child patients (2, 5, 11).

Bennardo et al. (11) from Italy suggested that faculty must arrange the objectives, restructuring course components, and training on remote teaching for all the faculty members employed in pediatric dental education. They also suggested that the departments' Head encourage their faculty to adapt online teaching skills for use during times requiring modification to workflow. Deery (12) from the United Kingdom suggested that dental students might develop anxiety while adjusting to these new innovative teaching methods and students' anxiety management should be acknowledged with resources available. There is the necessity to re-form the education methods in pediatric dentistry to make it pivotal for inter-professional education. Online resources help supplement dental education in pediatric dentistry by various pediatric dental associations worldwide (Table 1). Many pediatric postgraduate programs hold online journal clubs, lectures, demonstrations, and online education modalities in pediatric dentistry open for all residents worldwide (Figure 1c). Virtual pediatric dental society conferences, webinars, and podcasts are available for dental students and residents (13), and these online resources are regularly available free to pediatric dental residents. The recent outbreak of COVID-19 gave a big challenge to the oral health care sectors, and the clinical practice and education system got massively affected. Subsequently, global health authorities impose new infection control protocols and sporadic orientations for the dental students to enhance their knowledge in a holistic way to assure a harmless atmosphere in their classrooms and dental clinics.

**Table 1:** Online resources for students to learn pediatric dentistry during COVID-19 from various pediatric dental associations and canceled meetings.

https://iapdworld.org/iapd-covid-19-resource-centre/ ^1^
https://www.aapd.org/about/about-aapd/news-room/covid-19/ ^2^
https://www.eapd.eu/index.php/subpost/information-about-covid-19 ^3^
**Canceled meetings**
• https://www.eapd.eu/index.php/subpost/update-for-the-15th-congress-of-the-european-academy-of-paediatric-dentistry-in-hamburg%2C-1-4-july-2020%2C-due-to-the-coronavirus-disease-covid-19^4^
• https://us.dental-tribune.com/news/aapd-2020-annual-session-canceled/^5^
• http://www.8meetings.com/2020/2020_pdaa.html^5^
• https://iapdsummit.org/^6^

The pandemic outbreak of COVID-19 has modified the training approaches (didactic and clinical) in dentistry for the subsequent years, intending to reduce transmission of COVID-19 infection (12, 14). There were cancellation of some conferences and postponement of some due to the present outbreak of COVID-19 disease (Table 1)^1^^,^ ^2^^,^ ^3^^,^ ^4^^,^ ^5^^,^ ^6^. However, the COVID-19 disease outbreak provided new paths for dental students' exposure to research. The majority of dental students and pediatric dentists have never witnessed such pandemic situations. World Health Organization (WHO) and the Centers for Disease Control and Prevention (CDC) and other professional societies, and local regulatory bodies succeeded in taking effective public health measures to benefit health care professionals and the population during this pandemic outbreak. Hence, these resources have grown in all dental education areas, including resource administration, disaster response, and coordination at institutional and departmental levels to face such pandemic situations. A recent American study^7^ (15) found that dental students are undergoing increased stress levels, and they felt that this COVID-19 outbreak affected their clinical learning. Recent insight from the United States of America, Brazil, and Australia (16) concluded that dental education and dental practices changed considerably because of the COVID-19 outbreak. The authors also recommended that researchers share their experiences on changes implemented and how they overcame challenges. Nuvvula and Mallineni (17) opined that pediatric teledentistry allows parental guidance, management plans, and follow-up with distant supported dental care and encouraged the virtual visits with the use of information technology for the children with dental needs prior to the physical appointments during the COVID-19 pandemic. A Brazilian study (18) reported that teledentistry is an alternative platform for dental support during these unusual times exemplified by the COVID-19 pandemic outbreak. The authors also opined that teledentistry allows children and parents to have telephonic or virtual engagements, with pediatric dentists providing triage and information on oral hygiene practices and instructions. Chang et al. (19) postulated that the present pandemic caused by the coronavirus had transformed the education system in dentistry. The authors cautioned the preparedness of dental educators to face the challenges. This review discusses the evolution of education in pediatric dentistry from classroom teaching to virtual teaching and the adoption of online education in pediatric dentistry through webinars. It also highlights the new strategies (Table 2) for students, academicians, and clinicians attending pediatric dentistry clinics during this pandemic suggested by various international associations.

**Table 2:** Suggested guidelines for encouraging dental student (undergraduate and postgraduate) participation in virtual consultations in pediatric dentistry.

• The postgraduate resident and attending pediatric dental faculty initiate the patient's video call. If permitted by the child and parent, the dental student then joins the video call.
• Postgraduate resident interviews with the child and parent while attending the pediatric dental faculty is present during the call and actively listening to the consultation. If permitted by the attending pediatric dentist, the dental student may also be involved with the child parent interview.
• Postgraduate resident explains the assessment and advice to the parent and child.
• The attending pediatric dental faculty may then provide added input or suggestions during the virtual video consult and answer all questions from the child or parent.
• The attending pediatric dental faculty, postgraduate student and dental student may discuss the case after the child and parent leaves the virtual video consult.

## Conclusion

The recent pandemic outbreak of COVID-19 had a significant impact on the dental education system. It is essential to understand the strategies to deal with the challenges associated with online teaching and video consultations. Teledentistry and online presentations such as lectures, demonstrations have proven to be successful alternate methods. Nonetheless, these important initiatives inspire dental students to leverage sluggish interests other than dentistry to form one-to-one support networks.

## Ethics Statement

The patients/participants provided their written informed consent to participate in this study.

## Author Contributions

All authors listed have made a substantial, direct and intellectual contribution to the work, and approved it for publication.

## Funding

The authors would like to thank the Deanship of Scientific Research at Majmaah University for supporting this work under project number No. R-2021-175.

## Conflict of Interest

The authors declare that the research was conducted in the absence of any commercial or financial relationships that could be construed as a potential conflict of interest.

## Publisher's Note

All claims expressed in this article are solely those of the authors and do not necessarily represent those of their affiliated organizations, or those of the publisher, the editors and the reviewers. Any product that may be evaluated in this article, or claim that may be made by its manufacturer, is not guaranteed or endorsed by the publisher.

## Footnotes

^1^Available online at: https://iapdworld.org/iapd-covid-19-resource-centre/ (accessed January 23, 2021).

^2^Available online at: https://www.aapd.org/about/about-aapd/news-room/covid-19/ (accessed January 23, 2021).

^3^Available online at: https://www.eapd.eu/index.php/subpost/information-about-covid-19 (accessed December 28, 2020).

^4^Available online at: https://www.eapd.eu/index.php/subpost/update-for-the-15th-congress-of-the-european-academy-of-paediatric-dentistry-in-hamburg%2C-1-4-july-2020%2C-due-to-the-coronavirus-disease-covid-19 (accessed on December 28, 2020).

^5^Available online at: https://us.dental-tribune.com/news/aapd-2020-annual-session-canceled/ (accessed February 3, 2021).

^6^Available online at: http://www.8meetings.com/2020/2020_pdaa.html (accessed June 24, 2021).

^7^Available online at: https://iapdsummit.org/ (accessed December 28, 2020).
